# Holding on or letting go? Patient experiences of control, context, and care in oral esketamine treatment for treatment-resistant depression: A qualitative study

**DOI:** 10.3389/fpsyt.2022.948115

**Published:** 2022-11-25

**Authors:** Joost J. Breeksema, Alistair Niemeijer, Bouwe Kuin, Jolien Veraart, Jeanine Kamphuis, Nina Schimmel, Wim van den Brink, Eric Vermetten, Robert Schoevers

**Affiliations:** ^1^Department of Psychiatry, University Medical Center Groningen, Groningen, Netherlands; ^2^Research School of Behavioural and Cognitive Neurosciences (BCN), University Medical Center Groningen, Groningen, Netherlands; ^3^Department of Psychiatry, Leiden University Medical Center, Leiden, Netherlands; ^4^Department of Care Ethics, University of Humanistic Studies, Utrecht, Netherlands; ^5^PsyQ Haaglanden, Parnassia Psychiatric Institute, The Hague, Netherlands; ^6^Department of Psychiatry, Amsterdam UMC Locatie AMC, University of Amsterdam, Amsterdam, Netherlands; ^7^Amsterdam Neuroscience, Research Program Compulsivity, Impulsivity & Attention, Amsterdam, Netherlands

**Keywords:** ketamine, treatment-resistant depression (TRD), patient experience, quality of care (QoC), set and setting, phenomenology, esketamine

## Abstract

**Background:**

Ketamine and its enantiomer esketamine represent promising new treatments for treatment-resistant depression (TRD). Esketamine induces acute, transient psychoactive effects. How patients perceive esketamine treatment, and which conditions facilitate optimal outcomes, remains poorly understood. Understanding patient perspectives on these phenomena is important to identify unmet needs, which can be used to improve (es)ketamine treatments.

**Aims:**

To explore the perspectives of TRD patients participating in “off label” oral esketamine treatment.

**Materials and methods:**

In-depth interviews were conducted with 17 patients (11 women) after a six-week, twice-weekly esketamine treatment program, and subsequently after six months of at-home use. Interviews explored participants’ perspectives, expectations, and experiences with esketamine treatment. Audio interviews were transcribed verbatim and analysed following an Interpretative Phenomenological Analysis (IPA) framework.

**Results:**

Key themes included overwhelming experiences; inadequate preparation; letting go of control; mood states influencing session experiences; presence and emotional support, and supportive settings. Patients’ attempts to let go and give into vs. attempts to maintain control over occasionally overwhelming experiences was a central theme. Multiple factors influenced patients’ ability to give into the experience and appeared to impact their mood and anxiety about future sessions, including level of preparation and education, physical and emotional support, and setting during the session.

**Conclusion:**

Better preparation beforehand, an optimized treatment setting, and emotional and psychological support during (es)ketamine sessions can help patients to “let go” and may lead to better quality of care and outcomes. Recommendations to improve quality of patient care in (es)ketamine treatment are provided, including suggestions for the training of nurses and other support staff.

## Introduction

In the last 20 years, intravenous (IV) infusions with ketamine or its left-turning enantiomer S-ketamine (esketamine) have shown rapid antidepressant effects in patients with major, often treatment-resistant depression (TRD) (1). Recent studies also suggest efficacy of other routes of esketamine administration such as oral (2–4) and intranasal esketamine treatment (5, 6). The latter is now a registered treatment for TRD. Evidence of efficacy has also been shown in a study in real world settings (7–9). The antidepressant effects of a single administration of ketamine are rapid and robust, yet transient, lasting an average of seven days (5, 10, 11), which is why many treatment programs offer repeated dosing. Ketamine acutely induces psychoactive effects often referred to as “dissociative” or “psychotomimetic” (12). The phenomenology of these effects overlaps with the psychedelic effects of classic serotonergic psychedelics (13, 14), including visual and auditory perceptual changes, altered proprioception, alterations in conscious states, experiencing detachment from the world or self, and mystical experiences (15–17). In psychedelic treatments, optimizing set (preparation, expectations, mood) and treatment setting (physical environment, support during sessions, use of music etc.) are thought to positively influence patient experiences and treatment outcomes (18–20). Whether this also applies to (es)ketamine treatment is unknown. Some therapists combine (es)ketamine with psychotherapy (21) but it is mostly administered as a pharmacological treatment and psychoactive effects are generally considered side effects (12, 22, 23). Patients with severe, treatment-resistant depression, particularly when compounded by other mental disorders, may have difficulty handling the potentially destabilizing psychoactive effects of ketamine, although there has been little investigation into their perspectives. Detailed knowledge about the relationship between acute psychoactive effects (including “dissociation”), therapeutic process and treatment outcomes is inconclusive (12, 22, 24). Oft-used psychometric scales–such as the CADSS–were developed to evaluate dissociation related to psychopathology, and may not be specific enough to assess dissociative experiences induced by ketamine, and its relation to depressive symptom relief. Moreover, there are no clear guidelines or recommendations for optimal patient care in (es)ketamine treatment (25), making it difficult for healthcare providers to know which elements contribute to effective treatment and constitute good quality care.

Recently, a number of qualitative studies investigated the experiences of patients undergoing IV ketamine treatment of alcohol use disorder (AUD) (16), suicidal ideation (26), major depressive disorder (MDD) (15, 17), and TRD (27–29). Most described the phenomenology of ketamine’s acute effects; other themes included adverse effects (26–29), perceived therapeutic mechanisms (16, 26, 29), patient attitudes, motivations, expectations (16, 27–29), and barriers and facilitators of (es)ketamine treatment (16, 29). With one exception (ketamine treatment of AUD) (16), most qualitative studies did not capture in detail how patients experience the ketamine treatment and which conditions facilitate optimal outcomes. Understanding patient perspectives on these phenomena is important to identify unmet needs, which can be used to improve (es)ketamine treatments. The current study was designed to investigate this knowledge gap.

## Materials and methods

### Design

This qualitative study was designed using an Interpretative Phenomenological Analysis (IPA) data collection and analysis framework (30, 31), using individual in-depth interviews to explore the experiences and perspectives of patients undergoing oral esketamine treatment for TRD. IPA is primarily concerned with understanding complex subjective phenomena, focusing on detailed descriptions of all aspects of respondents’ lived experience of a phenomenon, in this case oral esketamine treatment, that often remain under-examined in quantitative studies using questionnaires (32, 33). As such, sample sizes tend to be smaller than in quantitative research and are based on saturation of the information collected rather than statistical power. IPA puts emphasis on idiography, allowing one to fully explore individual perspectives first, before seeking to understand how these experiences converge and diverge within the group. Finally, our aim was to make sense of how patients interpret and understand their experiences of the esketamine treatment, which is a core element of IPA (31, 33, 34).

### Treatment setting

The off-label treatment regimen consisted of six weeks of twice-weekly, individually titrated dosages of oral esketamine provided to TRD patients at the department of psychiatry of the University Medical Center in Groningen and a specialized depression clinic in The Hague, Netherlands. Generic esketamine was administered in a liquid formulation; no patents or other commercial interests were involved. Key inclusion criteria were a DSM-5 diagnosis of MDD (based on the Mini International Neuropsychiatric Interview) (35), and being treatment-refractory defined as no sufficient response to adequate treatments with at least three different classes of registered antidepressants. Esketamine treatment was started at a dose of 0.5 or 1 mg/kg, increased with 0.5 mg/kg or less based on antidepressant effects and tolerability, with a maximum of 3 mg/kg, provided as add-on to standard antidepressant medications. The bioavailability of oral (es)ketamine is significantly lower than alternative routes, with studies estimating it to be between 8 and 24% of intravenous administration (36–39). For our oral off-label treatment, 0.25 mg/kg IV was taken as a common dosage (40), equivalent to between 1.04 and 3.13 mg/kg PO, which was the dose range investigated in the off-label treatment. During the initial six-week regimen, vital signs (BP and HR) were monitored before each esketamine administration, and at 30- and 120-min after ingestion of the medication. No other clinical contact took place during esketamine administration sessions. Most patients received esketamine in a private treatment room at the depression clinic or hospital; some received treatment together with one or two other patients in the same room. Patients who responded well to the initial six-week treatment were considered for continued outpatient “at-home” treatment, based on shared decision making with patients and clinicians, and safety assessment.

### Study participants

We used purposive sampling to recruit patients participating in off label oral esketamine treatment for TRD; treatment coordinators approached patients for participation in the current qualitative study. Seventeen individuals (11 women, 6 men; 8 from the Hague, 9 from Groningen) participated in a single open in-depth interview. Table 1 provides characteristics of our respondents.

**TABLE 1:** Respondent characteristics.

#	Sex	Age range	Diagnosis	Psychiatric comorbidity	Depression treatment history	# Esketamine sessions
P1	F	60–65	MDD	PD-NOS	- Multiple psychotherapies - Multiple ADs (SSRIs, SNRIs, TCAs)	15
P2	M	30–35	MDD	–	-Multiple ADs (SSRI, SNRI, MAOI, lithium), bupropion -ECT, rTMS	11
P3	F	45–50	MDD	APD PTSD symptoms	-Multiple psychotherapies -Multiple ADs (SSRIs, SNRI, TCAs, mirtazapine, lithium and quetiapine addition), bupropion -ECT	22
P4	F	35–40	BPD II	C-PTSD	-Multiple ADs (SSRIs, SNRIs, TCAs), bupropion, multiple mood stabilizers	62
P5	M	55–60	MDD	ASS	-Multiple ADs (SSRIs, SNRIs, TCAs, MAOIs, lithium addition, antipsychotics addition) -ECT	22
P6	F	40–45	MDD	–	-Multiple ADs (SSRIs, SNRIs, TCAs), bupropion -Multiple psychotherapies	11
P7	M	55–60	MDD	–	-Multiple ADs (SSRIs, SNRIs, TCAs, lithium addition) -Multiple psychotherapies	12
P8	M	40–45	BPD I	–	-Multiple ADs (SSRIs, SNRIs, TCA, lithium addition) -Multiple psychotherapies	16
P9	F	60–65	MDD	–	-Multiple ADs (SSRIs, SNRIs, TCA + lithium addition)	23
P10	F	50–55	MDD	ASS, PTSD	-Multiple ADs (SSRIs, SNRIs, TCA, MAOIs, lithium addition, antipsychotics addition) -EMDR, ECT	24
P11	F	60–65	MDD	–	-Multiple ADs (SSRIs, SNRIs, TCAs, MAOIs, lithium addition) -Multiple psychotherapies -ECT	316
P12	M	55–60	MDD	–	-Multiple ADs (SSRIs, SNRIs, TCAs, MAOIs, mirtazapine, lithium addition, antipsychotics addition), topiramate -EMDR	90
P13	F	60–65	MDD	–	-Multiple ADs (SSRIs, SNRIs, TCAs, MAOIs, lithium addition, antipsychotics addition) -Multiple psychotherapies -ECT	350
P14	F	50–55	MDD	–	-Multiple ADs (SSRIs, SNRIs, TCAs, MAOIs, lithium addition, antipsychotics addition) -Multiple psychotherapies	∼65
P15	M	40–45	BPD I	ADHD, Migraine headaches	-Multiple ADs (SSRIs, SNRIs, TCAs, MAOIs, mirtazapine, lithium addition, antipsychotics addition) -ECT, Light therapy	∼45
P16	F	40–45	MDD	ASS	-Multiple ADs (SSRIs, SNRIs, TCAs, MAOIs, lithium addition, antipsychotics addition) -Multiple psychotherapies -ECT, IV esketamine	28
P17	F	60–65	MDD	–	-Multiple ADs (SSRIs, SNRIs, TCAs, MAOIs, lithium addition, antipsychotics addition) -Multiple psychotherapies	12

ADs, antidepressants; ADHD, attention deficit hyperactivity disorder; APD, avoidant personality disorder; ASS, autism spectrum disorder; BPD, bipolar disorder; EMDR, eye movement desensitization and reprocessing; ECT, electroconvulsive therapy; MAOis, monoamine oxidase inhibitors; MDD, major depressive disorder; PD-NOS, personality disorder not otherwise specified; PTSD, post-traumatic stress disorder; SNRIs, serotonin-norepinephrine reuptake inhibitors; SSRIs, selective serotonin reuptake inhibitors; TCAs, tricyclic antidepressants.

### Data collection

Interviews were concluded shortly after the end of the initial six-week clinical treatment period to gauge patients’ impressions of the treatment as a whole, to explore their experience throughout the process, and to explore factors that may have benefitted or negatively impacted the experience. We also included several respondents who had at least several months experience of at-home esketamine use. Fourteen in-depth interviews were conducted by the first author (JB); the remaining three interviews by the third author (BK). Neither was involved in the treatment or had prior contact with respondents. Interviewers followed an interview guide, designed to inquire about participants’ perspectives, expectations, and experiences of the esketamine sessions and the treatment as a whole. The interview guide included open-ended questions e.g., “what were your expectations for this treatment?” or “how did you experience the treatment setting?”, intended to understand inductively how patients made sense of their experiences and the treatment context. See Supplementary material for the full interview guide. Interviews lasted between one and two hours (mean: 1 h 22 min). Due to varying Covid-19 related restrictions, 14 interviews were conducted *via* videoconferencing software; three were conducted face-to-face at an inpatient clinic. No noticeable differences were found in length or content of the different types of interviews.

### Data analysis

Audio-recorded interviews were transcribed verbatim. Transcripts were entered into MAXQDA, a computer assisted qualitative data analysis software to facilitate analysis of the interview transcripts. IPA (30–32) was used to guide the iterative analysis process; identifying patterns within and between patients’ experiences, and how patients make sense of and interpret their experiences (33). First, all transcripts were read by the first and third authors, allowing them to become thoroughly familiar with the content. Secondly, transcripts were analyzed independently. All authors read and analyzed several transcripts each, nothing comments, observations, and reflections. Third, individual analyses of the same transcripts were discussed between all authors until consensus emerged. Fourth, notes were rewritten into exploratory themes with a higher level of (psychological) abstraction. Fifth, after analyzing all cases independently, themes were grouped across transcripts, based on conceptual similarities, identifying both convergent themes and patterns, and divergent topics (where patient experiences differed) (41). Finally, these categories were re-examined and re-clustered, resulting in a list of major themes and sub-themes.

### Quality measures and scientific rigor

Several procedures were followed to ensure the validity and rigor of our findings. Using purposive sampling, we approached participants at both sites. This enabled us to describe the phenomenon in all its nuances, providing “thick” descriptions of participant experiences. To create an understanding of a specific situation, detailed information is always necessary. Through detailed, rich descriptions, we challenge readers to appreciate the persuasiveness of the researchers’ interpretations (42). Rather than using a predetermined sample size, we used data saturation to decide when to stop including new patients: i.e., when topics had been exhaustively explored and no new findings emerged. Throughout the analysis process, transcripts were discussed within the multidisciplinary research team (consisting of a philosopher, several psychiatrists, a medical ethicist, a psychologist, and a doctor-in-training) until consensus emerged, to triangulate the data and ensure validity. Finally, we followed the 21-item Standards for Reporting Qualitative Research (SRQR) and the 32-item COREQ (consolidated criteria for reporting qualitative research) checklist to ensure adherence to the highest methodological rigor (43, 44).

## Results

The following key themes were identified: overwhelming experiences; inadequate preparation; letting go of control; mood states influence sessions; presence and emotional support, and supportive settings.

### Overwhelming experiences

Throughout the treatment, patients reported a wide range of acute psychoactive effects, both positive and negative, during the esketamine dosing sessions. Some patients reported unexpected intense and occasionally overwhelming and frightening experiences during initial (low) doses, although for most patients these only occurred at higher doses. Descriptions repeatedly included (but were not limited to): bizarre, disorienting, a “rollercoaster ride”, dissociative (e.g., losing the concept of having a body), taking place outside time and space, entering “another world”, experiencing eternity, the void, relativity, and experiences of unity and connection with the universe. Experiences were sometimes described as spiritual, peaceful, and relaxing but often (also) as alienating, disorienting, or frightening.

*“At a certain moment in the clinic (…) I thought, I don’t know what is happening anymore (…) I had the feeling I was losing myself” (P14)*

### Inadequate preparation

Many patients expressed feeling unprepared for the esketamine sessions and insufficiently informed about the content and the intensity of the psychoactive effects of esketamine and emphasized the need for proper preparation. For example, one patient said that it was only after 2 years of esketamine treatment that someone told her that it could be beneficial to let go of control and be more relaxed. Seeing that those suggestions had a positive effect, she remarked: *“That’s when I thought, well yes, you could have said that much sooner” (P13).* A point of confusion was when patients were told that they should not expect any effects during the initial (low dose) sessions; whereas staff likely referred to the antidepressant effects of esketamine, patients took this to mean they would not experience any psychoactive effects in the first few sessions. And so, when patients did react strongly to a low dose during the first session(s), they were caught off guard and felt unprepared:

*“So, the first time (…) I got such a small cup to drink and I thought, I won’t notice anything until after six intakes. Within 5 min, everything started spinning and tingling [and became weird] so that scared me a lot. (…) I hadn’t anticipated this at all. I sat there pretty much by myself because the nurse had (…) left me alone.” (P6)*

#### Side effects or core treatment components?

During preparatory sessions, when staff did explain the potential psychoactive effects of esketamine, these were often referred to as “side effects.” Sometimes, staff members explicitly stated that these effects were not important for the treatment of their mood disorder.

*“I actually thought it was a little weird that they say it’s a “side effect,” because in my opinion it’s not a side effect but just how ketamine works. (…) When you take ketamine, this is what you feel.” (P10)*

Most patients, regardless of whether they viewed the subjective effects as pleasant or unpleasant, spontaneously referred to their experiences as “trips”; terminology not used by the interviewers or staff. Some respondents thought these trips were part of the therapeutic process and valuable, at the same time expressing that staff did not seem to agree; other respondents thought the subjective “side” effects were irrelevant for the treatment.

*“I personally think (…) that the trip is the most important thing. If I have had a trip, I can function much better the same day and the following days before I get another dose. (…) My idea is that during the trip you unconsciously solve things in your head, that you come to certain insights (…) which helps you without being aware of it.” (P15)*

#### Hope and expectations

Having looked up (es)ketamine treatment online or having read positive reports about (es)ketamine in the media, many patients described a mixture of moderate expectations and cautious hope that esketamine therapy would enable some positive change, “*some light at the end of the tunnel.*”

*“[The psychiatrist explained] that ketamine is not going to be the solution, it’s not going to get rid of my depression (…) But I do need to engage in something and to have prospects about the chance of improvement.” (P3)*

A number of patients, having exhausted all other conventional treatments (including electroconvulsive therapy or ECT), saw esketamine as their last treatment resort.

*“I had agreed with my psychiatrist that I would give it until my birthday; and [after] that we would initiate the euthanasia procedure.” (P11)*

### Letting go of control

A major theme that emerged throughout the interviews was patients’ attempts to either let go and give into, or to maintain control over their acute (sometimes intense) experiences and in relation to the treatment in general.

Control, and relinquishing control, was something that many patients struggled with, both in daily life and in the context of the esketamine treatment.

*“Letting go: it’s just a few letters, but it’s obviously very difficult [for me] (…) I don’t have that much to hold on to. So those little things that (…) you do have, you try to hold on to.” (P5)*

Practically all participants spontaneously described how being more or less successful at submitting to the experience influenced the content and their appraisal of the esketamine sessions and the treatment. Managing or failing to relinquish control was often mentioned in relation to the esketamine sessions themselves, which respondents described variously as overwhelming, confusing, or unfamiliar.

*“I found it a really unpleasant experience to be no longer present in my body and to give up control. That’s something I don’t like in any case, but it happened very violently there.” (P6)*

To maintain control over the psychoactive effects, respondents first tried resisting them. Most, however, acknowledged that attempting to prevent undesirable effects from occurring was counterproductive, and in fact generated stress, tension, and/or anxiety. This created a negative experience, reinforcing the idea that they had lost control over what happened to them.

*“The more frenetically you try not to think about something [negative], the more it forces itself on you, at least, that’s how I experience it.” (P9)*

Participants employed several strategies to avoid negative experiences during the esketamine sessions: actively avoiding difficult memories and dark thoughts; trying actively to think happy thoughts; trying to ignore the effects elicited by esketamine; focusing on something external (e.g., their hands, their phone, a clock); seeking to communicate with others; trying to control the flow and content of the experience; and forcibly trying to relax (as sometimes suggested by clinicians or relatives).

*“Letting go is just something I am very bad at. And if it’s something that I* must *do (…) well that doesn’t work [very well] if* at all! *[Relaxing more] wasn’t formulated as an obligation, but in my head it was.” (P6)*

For some patients, submitting to the treatment also meant temporarily casting off any ties to the outside world, and really engaging with therapy. For example, it was only after participant P1 stopped working (which she’d continued doing remotely during her hospitalization) that she experienced the full effects of the esketamine, both subjectively and therapeutically.

*“Well, the fact that I let [work] go for a while actually created room for the ketamine treatment. And also for feelings that come with that, and they’re not nice. [When I continued working] I could just get rid of those [feelings], and now I can’t” (P1)*

Many patients struggled on and off with letting go throughout their treatment course; sometimes even within the same session, as exemplified by this respondent:

*“After about fifteen minutes you disappear into another world. You get sucked into it. But to allow that [experience] you have to keep your eyes closed. And when you open your eyes again, [you’re back in] your room. Every time [I did that] there was doubt: Do I have to, can I allow it, can I close my eyes again?” (P5)*

When patients did manage to reduce their resistance, accepting rather than avoiding the experience, it often became less jarring, erratic, or anxious and instead became calmer, smoother, and more pleasant. These “smoother” experiences often also contributed to a more positive affect directly afterward. Ceding control appeared not only a requirement for positive experiences, but patients also considered this an intrinsically positive, and sometimes meaningful or therapeutic experience.

### Mood states influencing session experience

Several patients mentioned that, for various external reasons, the beginning of their treatment period was chaotic or unclear. This created a state of restlessness and unease, which amplified anxiety and nervousness that some patients already felt before starting this treatment; this mood often carried over to the esketamine sessions. Then, when patients had (unexpectedly) frightening or overwhelming experiences, experienced as a loss of control, this triggered or further exacerbated their anxiety.

*“[Earlier] I would become really very anxious and, yes, then you lose control. And that’s what I would change in the treatment. To [tell] people who get [ketamine] for the first time: let it come over you. And really discuss it consciously with [patients]. Because I’ve actually had some unjustified fears as a result of [my initial frightening experiences]” (P1)*

Unpleasant experiences during (early) esketamine sessions often stayed with patients, and negatively impacted subsequent sessions.

*“Every time I take ketamine, I’m still experiencing this aversion against it. That is difficult and I’m still searching for a way to cope with that… it gets better, but that one time had a big influence” (P11)*

Patients also described what was helpful in the phase before esketamine sessions: starting sessions quietly, in a clear, calm, open state of mind, and letting go of specific goals or expectations regarding the sessions. Trying to maintain control over the content or outcome of the sessions and having fixed expectations often ended up being counter-productive; some patients expressed becoming disappointed after their expectations had not been met, which in turn negatively impacted their mood.

*“The more I focus on the trip, the more I look forward to it, the more it doesn’t come. (…) The doctors explained to me that ketamine works even if you don’t have a trip but to my mind that was not the case. [So, whenever I did not have a trip] I got so disappointed that I immediately became more depressed again.”* (P16)

### Presence and emotional support

The degree to which patients felt supported, both professionally (by nurses and other clinical staff) and informally (by relatives, partners, or friends) was an important factor in patients’ ability to give into the esketamine-experience. Thematically, support referred to both physical presence, and emotional and psychological support.

#### Supportive presence

All patients expressed the need for support through physical presence, i.e., not feeling left alone during the (first) esketamine sessions. This was especially noticeable in patients who were anxious, nervous, or expressed difficulty giving into the treatment. Patients mostly felt supported by nurses, or stated that it was enough to know that nurses were available if needed. However, some respondents mentioned that, particularly during the first sessions, nurses left after administering esketamine, returning regularly to check in (and administer blood pressure checks or questionnaires). For them, being left alone reinforced feelings of anxiety, which also carried over to subsequent sessions and hindered their further ability to give into the experience.

*“For people like me, who find it difficult to let go (…) maybe it would have been good if they had said ‘you know, the first time I’ll stay with you. Just try to go in calmly, and I’ll sit here, if there is anything [you need]”. That might have given some peace of mind. (P1)*

The need for support was particularly strong during the early stages of the treatment, when everything was new and unfamiliar, and patients were not sure what to expect. Being present, providing reassurance, and occasionally holding patients’ hands were seen as very comforting and supportive, and for some patients helped maintain a connection to (bodily) reality. Patients often said that the presence of their partner, relative or someone else close to them was very important.

*“The few times [I managed “to surf the waves”] were the times my husband was there. (…) [Having] someone who I trust completely sit next to me [ensured] I could also think of it a bit more like an adventure (…) instead of just resisting it” (P6)*

While this link to reality helped reduce some patients’ anxiety, sometimes it prevented them from becoming fully immersed in the experience.

#### Emotional support and trust

Feeling left alone and unsupported was not only related to the physical absence of nurses. Many patients also mentioned the need for emotional support by someone empathetic, whom they trust, and/or had previously established rapport with.

*“Contact [with the nurses] remained very superficial and very brief (…) I know they are busy and cannot hold my hand for three hours, but I found this very meagre. I felt very left alone. (…) [I missed] the feeling that someone was watching me.” (P6)*

Having someone present during the session who takes their (frequently described as bizarre) experiences seriously was important; while nurses were available, not all seemed to always understand patients or have the time to patiently and actively listen to their stories. Several patients suggested that the staff ought to have self-experience with (es)ketamine: to understand how their presence and attitude affects patients, and to help patients convey experiences that were sometimes ineffable.

*“[The nurses] don’t know. They can be nice, they can take care of you, they can offer you security, which they do very well, but they don’t know what you are going through. Unless they have used [ketamine] themselves.” (P1)*

#### Support with integration

Other reasons why patients thought it important to share their experiences was so they could recollect the ephemeral content of the experience longer, and to help them make sense of their experiences. Some participants shared their experiences with other patients, with partners or relatives, or in the example below, with their pastor.

*“I find it very difficult to share my experience [of being in heaven] because others cannot fathom what it is like. That is why I [said]: everyone should have experienced what it is like at least once.” (P16)*

Some patients considered parallel treatment trajectories with other patients helpful, as it allowed them to share and compare their experiences. Sharing also helped with sense-making and to know they were not alone in experiencing this, although not all patients felt this way. Several participants remarked that having a mental health professional to discuss or reflect upon their experiences would have been useful.

Certain patients wrote down insights, reflections, and experiences immediately after the esketamine session to regain the memory of their acute experiences, which were described as fleeting and often lasting no longer than the day of the session itself. After the sessions, several respondents said it helped them to take the rest of the day off, to rest, take time for themselves, and to process and integrate their experiences.

### Supportive settings

Different elements in the treatment environment contributed to patients’ (in)ability to give into the experience.

#### (Lack of) privacy and silence

**FIGURE 1:**
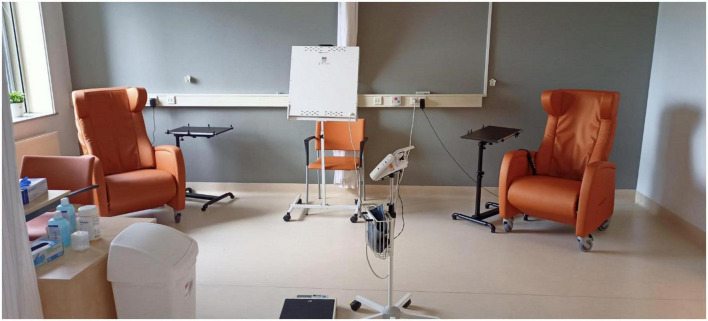
Room at the outpatient clinic (UMCG) used for (occasionally parallel) esketamine sessions.

**FIGURE 2:**
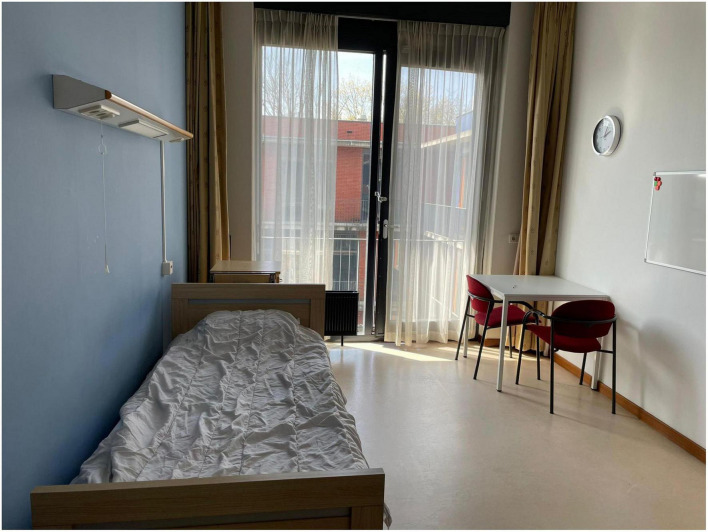
Typical treatment room at the inpatient clinic (The Hague) also used for esketamine sessions.

At the outpatient clinic, some patients received esketamine together with other patients in the same room (see Figure 1). In the inpatient clinic, patients received esketamine in their own room (see Figure 2). Esketamine enhanced sensory input (particularly sounds), and patients reported being easily disturbed by the presence and noises made by other patients (some of whom became quite agitated), their companions (e.g., partners, family members), and nurses.

*“Everything is so much more intense because of the ketamine, so you can hear sounds much better (…) and then it becomes quite unsettling.” (P4)*

One patient started her sessions later than two other patients (taking her hypertension medication beforehand); as a result, at her peak intensity other patients already started talking.

*“That was very difficult (…) as I am very easily distracted, and very sensitive to sound (…) [One time] a new patient and his wife just turned on the radio. Even my friend who was with me, was like: gosh, should I say something?” (P17)*

Most participants agreed that the presence of others was quite disruptive to their experience: *“You just hear it when someone comes in, even if you’re [deep] in a trip” (P4)* Esketamine put them in quite a “vulnerable” state, and interruptions by nurses entering and leaving, undergoing blood pressure checks (at 30 min, often at the height of the intensity of the esketamine experience), and filling out (“ill-timed, inappropriate, unsuitable, generic”) questionnaires were considered a source of nuisance and unrest. Another source of uncertainty for patients was not knowing whether they needed to communicate or interact with nurses, which hindered their ability to surrender to the experience.

*“When someone enters, you should only have to raise a finger. That’s what I do with [my nurse] (…). If I raise my thumb, he leaves again and then I stay in the trip (…) But I shouldn’t have to answer. (P1)*

Overall, most participants endorsed the value of privacy, due to the fragility of the esketamine induced state, the enhanced sensitivity to sound and the fact that interruptions hindered their ability to become fully absorbed in the experience. As one respondent remarked: *“if I know someone’s going to be watching, like the nurse or something, I stay a lot more alert”.* Similar remarks regarding privacy were made by patients who took their esketamine at home:

*“The only difference with the clinic is that at home I put in earplugs, so I really feel shut off from the world. In the clinic someone came to check on me every so often and I found that very disturbing. And I don’t have that at home, so I find that very pleasant. My phone goes on silent and then [with earplugs in] I’m not disturbed by anything or anyone.” (P16)*

In addition to external distractions, internal distractions (e.g., preoccupations, to-do-lists, recent conversations) also diverted patients from their internal experience.

*“For example, last week I was not feeling so well. I was just busy, you know, a child at home all day (…) a number of appointments, my wife who has to work more (…) My head just washes over, then I can’t organize anymore and my mood deteriorates.” (P15)*

#### A warm, comfortable environment

Commenting on the physical environment in which esketamine sessions took place, respondents found the clinics too “clinical and sterile” and inconsistent with their internal experience:

“*It is such a hospital affair; those curtains were very much hospital curtains. But luckily (…) if you turned the chair around you could look outside. I found that much more pleasant.” (P8)*

The ideal setting, described by patients, was somewhere that feels safe, that is warm and comfortable, where they can lie down, and where they don’t have to worry about being disturbed by others.

In addition to the physical environment, patients also mentioned the importance of structure around the esketamine sessions. Whereas some appreciated the structure and clarity offered by the clinic, others preferred a more flexible and outpatient treatment. For some, the lack of structure at home contrasted negatively with the highly structured environment of the clinical esketamine treatment, where esketamine sessions took place at a set time, and was unencumbered by patients’ daily chores and household interactions.

*“Now that I also have experience taking it at home, you’d almost say, you must take this in a clinic. It’s not just the ketamine, but it’s also being detached from your own world. Your home, your kids, your girlfriend, everything. You go [to the clinic], take your ketamine, and just live… in a world where (…) all you have to do is be with yourself.” (P5)*

#### Rituals and strategies to optimize effects

Creating the ideal setting was a process of trial and error for most patients who took esketamine at-home. Lacking clear instructions on how to take esketamine at home, most patients started by following the clinic regimen and timing. Over time, becoming more familiar with the effects of esketamine, they developed different strategies to optimize esketamine’s effects, such as by shaping their home environment to suit their preferences, and by developing their own rituals. Contrasting the clinic with their home setting, respondents construed their ideal setting: “When I took it at home, I had control over my environment.” (P8)

*“I lie down either on the couch or on the bed. (…) In summer the light shines in the bedroom and that’s pleasant. So I try to find the place where I feel as comfortable as possible.” (P11)*

For some, this meant altering the timing of esketamine intake, based on how it affected them acutely and throughout the remainder of the day afterward. Some started taking esketamine before bedtime in order to minimize or avoid any (unpleasant) subjective effects, or the impact of the tiredness following esketamine sessions. By contrast, others took esketamine early in the morning: both to circumvent disturbances and to ensure his whole day is not occupied by the esketamine session.

*“I get up at 4 a.m. and then I have a whole ritual drinking coffee and smoking a cigarette and when that is finished, usually an hour later, I take the ketamine and then I have over two hours (…) to relax without having people walk around me or talking. So I create my own safe bubble in that moment (…) I really try to create a relaxed atmosphere for myself.”* (P15)

Other strategies included eating beforehand, writing down all worrying thoughts beforehand, asking friends to check in regularly, and planning nothing on esketamine days:

*“Monday and Friday are just the days when I am not available and do not schedule appointments, and that gives a certain peace of mind.”* (P16)

Participants also mentioned other effective strategies or interventions that they discovered along the way, frequently wishing they had known these before starting the treatment. Some used breathing and mindfulness exercises: beforehand to go into sessions feeling calm, and during, to maintain some sense of control. Respondents also mentioned that having practical suggestions, to help them to let go of control and give into the experience, would have been very useful in the preparation phase.

*“In the beginning (…) I was very restless when I went in [the ketamine session]. Sometimes I went in crying. Or angry. But now that I have those breathing exercises–which I practiced with two nurses here–I’m not so heavy-handed anymore. Maybe a little sensitive, sad, but not so explosive.”* (P1)

#### The role of music

Looking for ways to shut out outside noise, some respondents used ear plugs or listened to music. The optional use of music was not often suggested by clinical staff. In addition to dampening intrusive external noises, music had several other uses. For some, music acted as an anchor point to reality, by providing structure and helping participants keep a grip on time (particularly in the case of familiar songs). This was a point of ambivalence for participant 2, who said that he stopped using music when he realized that it kept him too attached to the outside world: “*not that it’s wrong or anything, but (…) it very much kept me grounded”*.

Indeed, some patients remarked that music helped them to focus on their internal experience, allowing them go deeper into the experience. For some, music provided (emotional or psychological) direction or depth to the experience, and made it less jarring and more fluid for some.

*“Music just does a lot for me. (…) On both sides: I am not too afraid to lose control, plus I can enjoy my music.” (P13)*

Music types found to be supportive and which helped respondents enter into the experience more easily included calming, light (classical) music or repetitive songs; some used music that fit their mood at the time of the session, or songs that were personally meaningful. Participant 16, for example, listened to personally meaningful mantras (phrases that are repeated over and over) that helped increase the emotional depth of the experience:

“*When it hits you, yes, I notice that I feel tears rolling down my cheeks. Not because I feel so sad, but instead it’s very pleasant, in the sense that it touches you. (…) [Although] it can also evoke sadness. (…) But in general during a ketamine high, it’s deeply moving (…) and that’s what I really want to stay with me.”*

## Discussion

This qualitative study explored the experiences of patients participating in an “off-label” repeated oral esketamine treatment for treatment-resistant depression provided as inpatient, outpatient, and at-home treatment. Our phenomenological approach enabled us to explore in detail how individual patients perceived this treatment, what they experienced during esketamine sessions, how they made sense of their experiences, and where their experiences converged and diverged. We elucidated important treatment facets: the importance of proper preparation; letting go of control during overwhelming experiences; the impact of mood states on sessions; and the role of personal support and supportive settings. Despite a rapidly growing body of evidence on the use of various enantiomers and routes of administration of ketamine (45), non-pharmacological aspects of these treatments have rarely been discussed in the literature (16), contrasting with the central role of “set and setting” in treatments with classic psychedelics and MDMA (18, 20, 46).

### Letting go or losing control

“Letting go”, or patients’ attempts to either accept and surrender to or hold on and maintain control over their experiences was a central theme in our study. Another qualitative study on ketamine also noted that patients reported a loss of control but merely identified it as a “short feeling of being overwhelmed” followed by an “ability to go with it, or control it” (17). Our study suggests a more central role, where “letting go” is intricately linked to other major themes. It also showed that patients frequently struggled to let go of control, and that being unable to let go was associated with negative mood states, including an increase of (pre-existing) anxiety. Unpleasant experiences, in turn, negatively colored subsequent sessions, and made it more difficult for patients to undergo the esketamine sessions calmly and without resistance. When patients were able to “go with the flow,” the experience was often more pleasant and less dominated by anxiety; being able to relinquish control over the experience was an inherently meaningful experience for some. Understanding how to assist patients in this process is particularly important because the ability to adapt to unknown, uncertain, and unpredictable situations is typically impaired in patients with depression (47). In fact, impaired psychological flexibility has been proposed as a key *trans*-diagnostic trait underlying psychopathology more broadly, which has implications for the use of ketamine in other mental disorders (48).

### Preparation and education

One domain of the treatment in which this can be addressed, is during the preparatory phase. In therapeutic approaches with classic psychedelics, preparation entails educating patients about the possibility of challenging experiences, and instructing them to accept, rather than resist, whatever emerges, however difficult (46, 49–52). Supporting TRD patients to go along with potentially difficult and overwhelming experiences, instead of avoiding them, can promote patient care and comfort by reducing distress (53, 54). The shift from avoidance/control to acceptance/surrender has been suggested as an important therapeutic mediator in treatment with classical psychedelics (51–53, 55, 56); further research should investigate whether this process holds therapeutic promise for (es)ketamine treatment as well.

Consistent with other qualitative studies on ketamine treatment (26–29), respondents were often overwhelmed by the “side effects” of esketamine. Using this specific terminology for the acute psychoactive effects of ketamine implied that these are undesirable, reducing the likelihood for patients to recognize and interpret such experiences as potentially therapeutic, and of sharing their experiences with staff afterward, particularly since some participants in our study suggested their subjective experiences were therapeutically valuable. Irrespective of whether they contribute to better outcomes, fewer negative experiences may lead to reduced dropouts. Further, maintaining a balanced perspective on esketamine is also important to manage expectations, particularly in the light of the overly positive media coverage of (es)ketamine as a novel treatment for depression (57, 58) and to avoid disillusionment of this fragile patient population, many of whom may perceive (es)ketamine treatment as a last resort [see also (29)]; some respondents were in the advanced stages of the required evaluation and consultation procedure for euthanasia; in Netherlands this possibility exists for some exceptional untreatable cases (59).

### Common factors of esketamine treatment

Finally, we identified several elements of “setting” that facilitated or hindered patients’ ability to give into the experience, that are broadly consonant with the common factors theory in psychotherapy and insights from psychedelic therapies [see Gukasyan and Nayak (60) for an excellent discussion]. These factors include (a) presence and emotional support (an “emotionally charged, confiding relationship with a helping person”); (b) supportive settings (“a healing environment”); (c) framing and preparation for the psychoactive action of (es)ketamine (“a rationale or conceptual scheme providing a plausible explanation for the patient’s suffering and a means of alleviation”); and (d) rituals and strategies to optimize effects (“a ritual that requires participation of both patient and therapist that is mutually believed to be the means of succor”). Qualitative studies in other settings (e.g., Intensive Care Units) have also found that (unexpected) noise and interruptions have a negative impact on patients’ health and well-being (61), further emphasizing the importance of conducting (es)ketamine treatment in calm, low-stimulus, intrusion-free environments, especially because (es)ketamine is known to heighten sensitivity to sound (62). Our findings also corroborate two other qualitative studies into ketamine treatment that emphasized the importance of rapport with staff and a comforting environment (16, 29). Over time, patients taking esketamine at home spontaneously altered the environment and timing of intake to better suit these needs. Finally, mindfulness, breathing exercises, and music are simple interventions that can empower and promote a sense of agency in patients by providing tools that enable them to more easily accept or cope with the effects of esketamine. Music remains an underexplored tool in (es)ketamine treatment, as it can be used to dampen external noise, provide grounding, and increase emotional depth and meaning (63, 64).

Notably, a recently published expert opinion makes scant mention of set and setting in their implementation guidelines, apart from recommending a “comfortable and adaptable environment.” The authors do not provide specific recommendations on what such an environment should look like beyond emphasizing physical and psychiatric safety (45). The provision of psychological or emotional support before, during and after the sessions is not mentioned, which likely represents the default perspective on (es)ketamine treatment. Treatment providers involved in the off-label esketamine treatment in the current study were surprised to hear that their patients had often had difficult experiences. Despite systematic evaluation with standardized questionnaires (e.g., DSS, SAFTEE, IDS-SR), clinicians were largely unaware which elements of the treatment were specifically (dis)agreeable to patients. This is partly explained by the inadequacy of standardized questionnaires, which cannot account for individual variances and which are insufficiently fine-grained to capture the full spectrum of patients’ experiences (15, 22). Also, without explicit, open inquiry about their experiences, patients may not be likely to spontaneously divulge challenging experiences. At both study sites, after sharing our preliminary findings, changes have been implemented in the way patients are educated about esketamine beforehand, limiting sessions with multiple patients in one room, instructing staff to build interpersonal trust with patients, and to remain with them during esketamine sessions for as long as needed. Without in-depth qualitative interviews this important information would have been missed, and therefore this study has already made a tangible impact on improving the overall patient care. We have summarized our most important practical recommendations in Table 2.

**TABLE 2:** Recommendations aimed at improving patient safety and care in trials or off-label treatment with (es)ketamine.

Preparation	
Instructions	Provide clear instructions on timing, nature, intensity, and unpredictability of a wide range of subjective effects
Neutral terminology	Use neutral terminology rather than verbiage with negative associations (“side effects”). It can be useful to remind patients to keep an open mind regarding any potential therapeutic effects
Promote acceptance	Instruct patients about the potential “loss of control” during the acute phase, and the value of accepting difficult emotions, thoughts, memories rather than resisting these
Calming techniques	Instruct and practice simple techniques related to surrendering to the experience: mindfulness or breathing exercises, hand holding etc.
Calm, open-minded	Support patients to enter each session calmly, and with a clear and open mind
Minimize anxiety	Remind patients about the transient nature of (negative) experiences, and the fact that they’re in safe hands and in a safe environment
Expectation management	Manage/minimize expectations about the content of the acute ketamine sessions, and about any potential outcomes of the treatment

**Support**	
Therapeutic rapport	Establish rapport with staff or others who will be present during and after dosing sessions
Physical presence	Ensure physical presence of nursing staff, particularly during early (low-dose) sessions, and upon patient request
Partners or relatives	Partners, relatives or friends can be a source of comfort and calm during sessions (both clinically and for at-home use)
Debriefing/integration	Availability of clinical stuff to help patients debrief, remember fleeting experiences, and discuss potentially transformative or overwhelming experiences
Hand holding/touch	Holding hands can be reassuring when patients experience distress or anxiety (however: always discuss beforehand)
Validation	Take experiences seriously, either through integratory talks or by facilitating interaction or discussion with other patients

**Setting**	
Music	Offer the use of calming music, and the possibility of (noise cancelling) headphones
Privacy, stimulus-free	Ensure privacy, minimize interruptions, and measurements around dosing sessions
Comfort	Provide a warm, comfortable environment (blankets, pleasant surroundings, possibility to lay down, etc.)
Optimizing home setting	Provide suggestions on ideal home setting (e.g., privacy, reducing external stimuli, avoiding distractions by others, the use of music, suggesting strategies on mitigating undesirable effects; acutely, post-acutely, and over the next days)
	

### Strengths and limitations

This study had both strengths and limitations. Using a phenomenological approach enabled us to access patients’ subjective experiences, yielding rich descriptions, and providing important insights into treatment variables and patients’ lived experiences not captured by standardized questionnaires. Often used psychometric scales such as the Clinician-Administered Dissociative States Scale (CADSS) and the Dissociative Experiences Scale (DES-II) were developed to evaluate dissociative experience as part of psychopathology and not designed to study or measure the specific type of dissociation as induced by ketamine nor to provide insight into potential therapeutic mechanisms. Further, this study addressed non-pharmacological treatment aspects regarding set and setting that, although generally recognized as important factors in determining patient care, are understudied in ketamine research. This directly led to adjustments in how esketamine treatment was offered for this population. This study was conducted on oral esketamine; we currently do not know whether other administration methods or enantiomers have similar or distinct effects; future studies should also investigate whether other approaches are more suitable for such treatments (65). A limitation was that we only interviewed patients who finished their initial six-week treatment. This may lead to response bias, as patients who discontinued esketamine treatment because of limited or even negative clinical effects were not included. Yet, this was a “last resort” treatment for many patients, which meant that they likely continued their treatment for at least the full six-week regimen given the lack of viable treatment alternatives. Moreover, we received a mix of positive and negative experiences of the esketamine treatment, reducing the likelihood of selection bias. It is possible that our participants experienced more difficulties with this treatment than other TRD patients since no consensus exists regarding the definition of TRD (66, 67). In the current study, TRD was defined as insufficient response to adequate treatments with at least three different classes of registered antidepressants. In fact, respondents had tried multiple (classes of) antidepressants, as well as augmentation medications, multiple psychotherapies, and often other proven-effective interventions such as rTMS or ECT as well. Thus our study population is a group of bonafide TRD patients, with a level of treatment resistance that was significantly higher than in some other studies. The sample size of the study population could raise questions about (external) validity of its results: nonetheless 17 respondents is a considerable sample size in qualitative research. Phenomenological research seeks to develop a contextual and layered understanding of patients’ lived experiences, instead of non-contextual generalizability. This does not mean that IPA lacks generalizability, but rather that theoretical generalization must be separated from statistical significance (68). IPA is a rigorous methodology, which requires constant methodological reflexivity of all researchers involved. Finally, due to the phenomenological nature of our study we focused on the extent of variation in which the observed situations occurred, and how exemplary these situations were, rather than their statistical significance (69, 70).

## Conclusion

Our qualitative study demonstrates that specific elements of set (preparing patients, offering reassurance, minimizing anxiety, instilling confidence, promoting agency) and setting (a warm, comfortable, silent, and private environment, with physical, interpersonal, and empathetic professional or informal support) are important determinants of quality of care, even when esketamine is provided as a purely pharmacological intervention. Although our study investigated the perspectives of TRD patients in “off label” oral esketamine treatment, it is tenable that the experiences described in our study are also applicable in other ketamine treatment approaches, trial designs, countries, and for patients suffering from other (mood) disorders. We therefore propose that future (es)ketamine treatments consider implementing our recommendations in order to improve the quality of patient care in (es)ketamine treatments, and to more rigorously study patient experiences using a combination of quantitative and qualitative methods.

## Data availability statement

The raw data supporting the conclusions of this article will be made available by the authors, without undue reservation.

## Ethics statement

Ethical approval was not required for this study in accordance with the review by the Medical Ethics Review Board of the University Medical Center Groningen (METc UMCG). Written informed consent to participate in this study was provided by the participants. Written informed consent from the individuals for the publication of any potentially identifiable images or data included in this article was obtained.

## Author contributions

JB and RS conceived of and designed the qualitative study. JB and BK collected the data. JB conducted 14 interviews. BK conducted 3 interviews. JB conducted qualitative analysis and in part by BK, NS, WB, and RS. All other authors contributed to the writing of the manuscript and approved the final manuscript.
