# Supplementary material for: Holding on or letting go? Patient experiences of control, context, and care in oral esketamine treatment for treatment-resistant depression: A qualitative study
Source: Front Psychiatry. 2022 Nov 25;13:948115. doi: 10.3389/fpsyt.2022.948115 (PMC9732097; doi:10.3389/fpsyt.2022.948115)
Supplement: Supplementary file 1 [file Data_Sheet_1.docx]

Interview guide oral esketamine TRD

**Examples of clarification questions**

- Can you tell me more about this?

- Can you tell me anything else?

- Can you give an example?

First of all, would you like to tell me something about yourself? How would you describe yourself? (

How did you come to participate in the ketamine treatment? (Prompt: what were your reasons to participate?)

Can you describe how you felt before participating in this study?

What were your expectations of this study / treatment?

Can you describe what happened during the ketamine sessions? (E.g. how did you first notice that ketamine did something? Prompt: emotions, memories, insights, feelings, internal, thoughts, visions, important themes)

How did you experience the treatment setting? (Prompt: safety, treatment room, preparation, emotional safety, therapists, both positive and negative)

How did these affect your experience during the ketamine sessions?

How did you feel immediately after the session was over? And in the days / weeks afterwards? (Prompt: did you feel better, worse, calmer, more restless?)

How is it to give the experiences of the ketamine sessions a place (in your daily life)?

What is it like to talk to others about your experiences of this treatment? (Prompt: partners, family, friends, colleagues)

What do you notice about the effect of the treatment? (Prompt: can you say something about impact of treatment? only positive or negative? What do partners/friends/family say? [sleep/nightmares, emotions, meaningfulness, creativity, relationships with family/friends, work, hobbies, drinking, medications, drug use, nature, activities])

Looking at yourself, what do you see as the most significant changes compared to before this study began?

Can you explain in your own words how you think this treatment works?

If you had the opportunity to change anything about the treatment, what would you do differently? (Prompt: would you recommend this treatment to others who are in a similar position?)

Is there anything else you would like to share?
